# Dietary Supplementation with Raspberry or Strawberry Seed Oil Impacts Folliculogenesis, Hormonal Parameters and the Fatty Acid Profile in the Juvenile Rabbit Ovary

**DOI:** 10.3390/ani16101528

**Published:** 2026-05-16

**Authors:** Małgorzata Grzesiak, Katarzyna Michta, Kalina Galińska, Michał Kmiecik, Sylwia Pałka

**Affiliations:** 1 Department of Endocrinology, Institute of Zoology and Biomedical Research, Faculty of Biology, Jagiellonian University, Gronostajowa 9, 30-387 Krakow, Poland; kasia.michta1999@gmail.com (K.M.); kalina.galinska@student.uj.edu.pl (K.G.); 2 Department of Genetics, Animal Breeding and Ethology, University of Agriculture in Krakow, Al. Mickiewicza 24/28, 30-059 Krakow, Poland; michal.kmiecik@urk.edu.pl (M.K.); sylwia.palka@urk.edu.pl (S.P.)

**Keywords:** raspberry seed oil, strawberry seed oil, folliculogenesis, fatty acids, ovary, rabbit

## Abstract

This study demonstrated the effects of dietary supplementation with 1% raspberry (RO) or 1% strawberry (SO) seed oil from 5 to 12 weeks of age (*n* = 6/group) on folliculogenesis, hormonal parameters, the ovarian fatty acid profile, and the expression of related genes in juvenile rabbits. After slaughter, ovaries and blood were collected. Ovaries were used for histology, fatty acid profiling, and gene expression analysis, while plasma was used to measure progesterone (P4), testosterone (T), estradiol-17β (E2), follicle-stimulating hormone (FSH), and anti-Müllerian hormone (AMH) concentrations. Both RO and SO reduced the number of primary follicles (*p* = 0.04), whereas RO increased the number of antral follicles (*p* = 0.04) compared with the control. In both supplemented groups, FSH (*p* = 0.04 and *p* = 0.035) and AMH (*p* = 0.04) concentrations were higher. RO increased P4 and E2 (*p* = 0.03 and *p* = 0.013) concentrations, while SO only increased P4 (*p* = 0.02) levels. SO altered the ovarian fatty acid profile, increasing selected monounsaturated fatty acids and reducing polyunsaturated fatty acids, likely by increasing the expression of the converting enzyme, stearoyl-CoA desaturase 5 (*p* = 0.038). Overall, both oils influenced folliculogenesis through hormonal changes, and SO modified ovarian fatty acid composition, which may affect ovarian function in juvenile rabbits.

## Simple Summary

Nutrition can influence female reproduction, but the effects of seed oils on the ovary are not well understood. This study investigated whether low dietary supplementation (1%) with raspberry (RO) or strawberry (SO) seed oils affects ovarian development in young rabbits. From 5 to 12 weeks of age, rabbits were fed a control diet or diets supplemented with RO or SO. After the feeding period, blood and ovarian samples were collected to examine follicle development, hormone levels, fatty acid profile, and the expression of genes related to lipid metabolism. Both seed oils reduced the number of primary follicles, while RO increased the number of antral follicles. Animals receiving the oils also showed higher levels of hormones, including the follicle-stimulating hormone and anti-Müllerian hormone. RO additionally increased progesterone and estradiol concentrations, whereas SO increased progesterone only. SO also changed the fatty acid composition of the ovary by increasing certain monounsaturated fatty acids and reducing polyunsaturated fatty acids, likely through changes in the expression of a lipid-metabolizing enzyme. Overall, the results suggest that dietary seed oils can influence ovarian development and hormonal regulation, which contributes to improving our understanding of how nutrition affects female reproductive functions.

## 1. Introduction

The ovary is a female reproductive organ that supports oocyte development and ovulation, and is also responsible for the synthesis of steroid hormones, namely progesterone (P4), testosterone (T), and estradiol-17β (E2), which regulate reproductive cycles and maintain endocrine homeostasis [1]. The progressive development of ovarian follicles from the primordial to preovulatory stage is called folliculogenesis. This process is strictly coordinated by interactions between endocrine, paracrine, and autocrine signals [2]. A crucial role is played by pituitary gonadotropins, e.g., follicle-stimulating hormone (FSH) and luteinizing hormone (LH), as well as local intraovarian mediators, including growth factors, cytokines, and metabolic substrates [3]. In addition, the steps of folliculogenesis are regulated by the anti-Müllerian hormone (AMH), which is produced in the ovary [4]. Rabbits are a useful model for studying the dynamics of ovarian follicle development because, in this species, folliculogenesis occurs entirely postnatally. During the first three weeks of life, the ovary contains primordial and developing follicles, while antral follicles emerge in juvenile animals around week 12 [5].

Besides internal signals, ovarian processes can be influenced by external factors, including fatty acids (FAs) derived from the diet [6]. FAs are not only a source of energy but also crucial components of biological membranes, as well as precursors for hormones and intracellular messengers [7]. Based on the presence of double bonds, they are classified into saturated fatty acids (SFAs, without double bonds), and unsaturated fatty acids (UFAs), which include monounsaturated fatty acids (MUFAs, with a single double bond) or polyunsaturated fatty acids (PUFAs, with multiple double bond) [8]. The limiting step in FA metabolism is the conversion of SFAs into MUFAs, which is carried out by stearoyl-CoA desaturases (SCDs) [9]. Ovarian cells can uptake FAs through the CD36 transporter, which has been identified in granulosa cell membranes of different species [6], following hydrolysis of triglycerides from circulating chylomicrons and very low-density lipoproteins by lipoprotein lipase (LPL) [10]. It is well established that FAs are involved in follicular development, steroidogenesis and oocyte maturation in both humans and animals [9,11,12]. On the other hand, certain pathological nutrition statuses, including obesity or high-fat diets, can affect the survival, proliferation, and energy balance of ovarian cells [13].

The growing interest in healthy diets has increased the consumption of fruit seed oils, which display health benefits attributed to their unique FA profiles and bioactive compounds, such as phytosterols, phenols, tocopherols carotenoids, and flavonoids [14]. Fruit seeds are the main by-product of fruit processing and are commonly regarded as waste. To facilitate their utilization and avoid disposal costs and environmental contamination, they are being increasingly used as a cheaper and alternative source of edible oils [15]. Recently, there has been growing interest in raspberry (*Rubus idaeus* L.) (RO) and strawberry (*Fragaria* × *ananassa*) (SO) seed oils, which are rich in UFAs, especially linoleic and oleic acids [16,17]. The direct impact of these oils on ovarian functions has not been studied. However, linoleic and oleic acids have been found to modulate ovarian follicle growth and development through the regulation of granulosa cell proliferation and apoptosis in humans and cows [18,19,20]. Furthermore, they affect ovarian steroidogenesis, namely P4 synthesis, in goats [21] and FSH-induced E2 production in buffalo [22]. Oleic acid has also been found to regulate the expression of genes related to FSH and LH signaling in bovine granulosa cells [23]. Although there is no data about the relationship between linoleic and oleic acid contents and AMH levels, one study showed that oleoylethanolamide (a bioactive lipid derived from oleic acid) decreased AMH concentrations in women with polycystic ovary syndrome [24]. Based on these findings, we hypothesized that low (1%) dietary supplementation with RO or SO would influence folliculogenesis, plasma concentrations of steroids, FSH, AMH, the FA profile, and the expression of FA metabolism-associated genes, namely *CD36*, *LPL* and *SCD5*, in the ovaries of 12-week-old rabbits. This work seeks to provide new insights into the role of fruit seed-derived oils in female reproductive biology, given that their supplementation could represent an effective strategy for regulating female reproductive potential.

## 2. Materials and Methods

### 2.1. Experiment Design

The animals used in this study were purchased from the Faculty of Animal Science of the University of Agricultural in Kraków (Poland). As previously described [16,17], Termond White female rabbits were kept with their litters in wooden cages placed in a temperature-controlled room until weaning, with unrestricted access to water, a 14 h light/10 h dark photoperiod, and mechanical ventilation. On postnatal day 35, the young were weaned and randomly assigned into experimental groups (*n* = 6/group). They were then transferred to metal cages intended for rabbit husbandry, each fitted with a plastic feeder and a nipple drinker.

Between 5 and 12 weeks of age, the rabbits had unrestricted access to pelleted feed provided by FHP Barbara Ltd. (Turza, Poland). In addition, the animals had unlimited access to fresh water, which was distributed via an automatically replenishing watering line. Animals in the control group received fresh non-supplemented pellets, while the other groups received pellets containing 1% RO or 1% SO (Table 1). The chemical composition of feed was performed by the laboratory of the Department of Nutrition and Fisheries (University of Agriculture in Kraków, Poland). Analyses were performed in accordance with internationally recognized methods (AOAC International, 2005) as follows: dry matter (method 934.01), ash (942.05), crude protein (976.05), crude fat (920.39) and crude fiber (978.10) [25]. We applied low supplementation levels that are sufficient to modify tissue fatty acid composition without altering energy balance as previously described [16,17]. The RO and SO were purchased from Olvita (Marcinowice, Poland). The oils were produced using cold pressing of oil seeds, which were then filtered and stored at 4 °C in dark glass bottles. Unrefined oils were mixed with the feed components, which were then pelleted by the feeder manufacturer. The FA profile of RO and SO were recently provided [16,17].

The rabbits were sacrificed at 12 weeks of age (day 84; *n* = 6/group; average body weight 2.6 kg; sexually immature) after a 24 h fasting period with unrestricted access to water. They were stunned, promptly exsanguinated, and subsequently skinned and eviscerated. Immediately after slaughter, the ovaries were harvested—one ovary was fixed in 10% buffered formalin for histology, while the contralateral ovary was snap-frozen for real-time PCR analysis and fatty acid content assessment. Blood samples were placed in heparin-coated tubes and centrifuged (4000× *g*, 10 min, 4 °C), and the plasma was stored at −20 °C until it was used for the hormonal analyses.

### 2.2. Ovarian Histology

The rabbit ovaries were fixed as previously described [27,28], and 5 μm thick sections were stained with hematoxylin–eosin (H&E). Follicle counts were performed in a blinded manner on three sections per ovary: one central section and two lateral sections, one on each side. The total follicle count was determined for each entire section. Follicles were categorized as follows: (1) primordial—surrounded by a partial or complete layer of flattened pre-granulosa cells; (2) primary—enclosed by a complete layer of cuboidal granulosa cells; (3) preantral—comprising two or more complete granulosa cell layers, a theca layer, and lacking an antrum; (4) antral—characterized by a fluid-filled antral cavity within the granulosa layers; (5) atretic—antral follicles exhibiting granulosa cells with pyknotic nuclei [27,28].

### 2.3. FSH and AMH Level Analysis

Plasma levels of FSH and AMH were measured using commercial enzyme-linked immunosorbent assay (ELISA) kits: Rabbit FSH ELISA Kit (# ELK5563; ELK Biotechnology, Denver, CO, USA) and Rabbit AMH ELISA Kit (#ELK9037; ELK Biotechnology, Denver, CO, USA), as well as a Labtech LT-4500 ELISA plate reader (Labtech International Ltd., Uckfield, UK) at 450 nm. The assay sensitivity was 0.68 mIU/mL for FSH and 25.4 pg/mL for AMH, with detection ranges of 1.57–100 mIU/mL and 78.13–5000 pg/mL, respectively. For both FSH and AMH, the intra-assay coefficients of variation were below 8%, while the inter-assay coefficients of variation were below 10%. All analyses were performed in triplicate.

### 2.4. Steroids Level Analysis

P4, T, and E2 plasma concentrations were assessed using commercial ELISA kits (#DE1561, #DE1559, #DEH3355, respectively; DRG MedTek, Warsaw, Poland) and a Labtech LT-4500 ELISA plate reader at 450 nm. The assay sensitivities were 0.045 ng/mL for P4, 0.083 ng/mL for T and 9.714 pg/mL for E2, with measurement ranges of 0–40 ng/mL, 0–16 ng/mL, and 0–2000 pg/mL, respectively. The intra- and inter-assay coefficients of variation were 5.4% and 9.96% for P4, 3.28% and 6.71% for T, and 3.1% and 4.7% for E2, respectively. All analyses were performed in triplicate.

### 2.5. FA Content Analysis

The FA content in the ovarian tissue was determined following extraction with a chloroform–methanol solution [29]. The FA composition of the corresponding methyl esters was analyzed by gas chromatography using a Trace GC Ultra system (Thermo Electron Corp., Waltham, MA, USA). Separation was achieved on a 30 cm Supelcowax capillary column (Bellefonte, PA, USA) with an internal diameter of 0.25 mm and a film thickness of 0.25 μm. Helium served as the carrier gas at a flow rate of 1 mL/min. The injector and detector temperatures were set at 220 °C and 250 °C, respectively. The oven temperature was held at 160 °C for 3 min, then increased at a rate of 3 °C per minute to 210 °C, and maintained at this temperature for 25 min. Individual FA methyl esters were identified by comparison to a standard mixture (Supelco 37 Component FAME Mix; Sigma-Aldrich, St. Louis, MO, USA) and CLA isomers (Sigma-Aldrich). All samples were analyzed in duplicate. The results are presented as a percentage (%) of the total FA content followed by calculation using ChromQuest 4.1 software (Thermo Electron, Milan, Italy).

### 2.6. Real-Time Quantitative PCR Analysis

Total RNA was extracted from frozen ovarian samples with TRI Reagent (Ambion, Austin, TX, USA) followed by RNA quality and quantity determination, and RNA integrity as described in detail [30]. The reverse transcription to cDNA was performed with a High-Capacity cDNA Reverse Transcription Kit (Applied Biosystems, Foster City, CA, USA), and subsequent real-time PCR was conducted using a StepOneTM Real-Time PCR System (Applied Biosystems) and the rabbit-specific TaqMan Gene Expression Assays (Applied Biosystems) for *CD36* (assay ID: Oc03395926_m1), *LPL* (Custom Plus TaqMan RNA Assay based on rabbit *LPL* cDNA sequence), and *SCD5* (Custom Plus TaqMan RNA Assay based on rabbit *SCD5* cDNA sequence). β-actin (*ACTB*; assay ID: Oc03824857_g1) was used as the endogenous control. All reactions were carried out in duplicates. A negative control without template was conducted, and genomic DNA amplification contamination was checked by omitting reverse transcriptase during the reverse transcription reaction.

The relative expression of *CD36*, *LPL,* and *SCD5* is presented as 2^−ΔCt^ values, which were used to statistically compare differences after normalization to *ACTB* levels (ΔCt value) [31].

### 2.7. Statistical Analysis

Data are presented as mean ± standard error of the mean (SEM) and were analyzed using Statistica v.13.1 (StatSoft, Inc., Tulsa, OK, USA). Normality was assessed with the Shapiro–Wilk and Lilliefors tests. Due to the lack of normal distribution of data, the Kruskal–Wallis test was performed, followed by Dunn’s post hoc multiple comparisons to evaluate differences between the control and supplemented groups. Statistical significance was set at *p* < 0.05 (95% confidence level).

## 3. Results

### 3.1. Effect of Dietary Supplementation with RO or SO on Ovarian Histology

**Figure 1:**
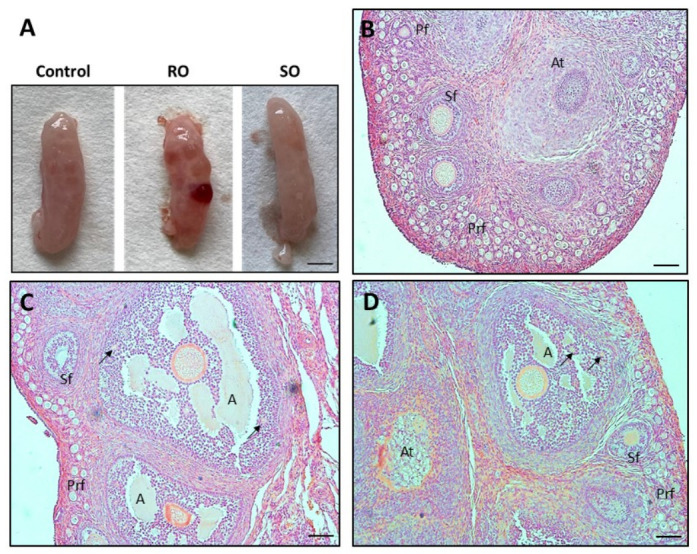
Representative photographs of the ovaries obtained from juvenile rabbits with visible antral follicles filled with follicular fluid. Bar = 10 mm (**A**). Histology of the ovaries from rabbits fed a control diet (**B**) or a diet supplemented with 1% RO (**C**) or 1% SO (**D**). Bar = 50 µm. A, antral follicle; At, atretic follicle; Prf, primordial follicle; Pf, primary follicle; RO, raspberry seed oil; Sf, secondary follicle; SO, strawberry seed oil; → Call-Exner bodies.

In all examined groups, primordial, primary, secondary, antral, and atretic follicles were observed (Figure 1B–D). There were significant differences in the number of primary and antral follicles following dietary supplementation with RO or SO: a decrease in the numbers of primary follicles was observed in both the RO (*p* = 0.04) and SO (*p* = 0.04) groups in comparison to the control group, while an increased number of antral follicles (*p* = 0.04) was only observed following RO supplementation (Table 2).

### 3.2. Effect of Dietary Supplementation with RO or SO on Plasma FSH, AMH and Steroid Concentrations

Dietary supplementation with RO and SO significantly increased FSH (*p* = 0.04 and *p* = 0.035, respectively) and AMH (*p* = 0.04 and *p* = 0.04, respectively) levels compared to the control diet (Table 3).

Regarding steroids, the RO and SO groups showed markedly increased plasma P4 concentrations (*p* = 0.03 and *p* = 0.02, respectively) in comparison to the control group. The E2 level was greater (*p* = 0.013) in the RO group than in the control and SO groups. The concentration of T was unchanged in both the RO and SO groups (Table 3).

### 3.3. Effect of Dietary Supplementation with RO or SO on FA Profile in Ovarian Tissue

Analysis of the FA profile in the ovarian tissue revealed pronounced changes following SO, but not RO, supplementation in comparison to the control diet (Table 4). Among SFAs, the lauric (*p* = 0.0097) and myristic (*p* = 0.005) acid contents were higher, while the amounts of stearic (*p* = 0.005) and arachidic acids (*p* = 0.049) were lower in comparison to the control group. Regarding MUFAs, the myristoleic (*p* = 0.005) and palmitoleic (*p* = 0.005) acid contents were greater than those of the control group. In addition, the level of cis-vaccenic acid was lower (*p* = 0.04) in the SO group compared to the RO group. Most PUFAs were decreased in the SO group compared to the control group, including dihomo-γ-linoleic acid (*p* = 0.018), arachidonic acid (*p* = 0.049), eicosapentaenoic acid (*p* = 0.049), adrenic acid (*p* = 0.007), and docosapentaenoic acid (*p* = 0.009). In contrast, the linoleic acid content was significantly higher (*p* = 0.018) than in the control group.

### 3.4. Effect of Dietary Supplementation with RO or SO on CD36, LPL, and SCD5 mRNA Abundance

**Figure 2:**
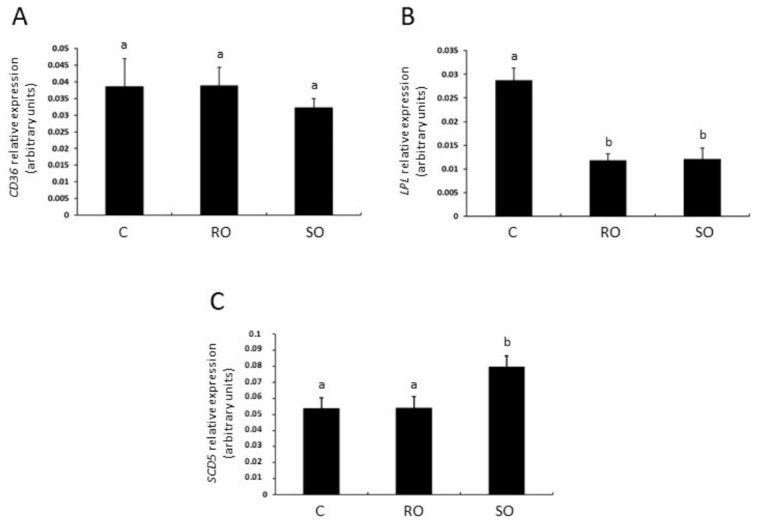
The mRNA transcript abundance of fatty acid translocase ((**A**); CD36), lipoprotein lipase ((**B**); LPL), and stearoyl-CoA desaturase 5 ((**C**); SCD5) in the ovaries of juvenile rabbits fed a control diet (**C**) or a diet supplemented with raspberry (RO) or strawberry (SO) seed oil. The mRNA abundance was determined using quantitative real-time PCR, and is expressed as 2^–ΔCt^ values relative to ACTB (β-actin). Different superscript letters denote statistically significant differences among groups (*p* < 0.05; Dunn’s multiple comparison test). *n* = 6/group.

To examine the effect of the RO- and SO-supplemented diets on the ovarian expression of the genes encoding FA translocase (CD36), lipoprotein lipase (LPL), and stearoyl-CoA desaturase 5 (SCD5), real-time PCR analysis was conducted. In both groups, *LPL* mRNA transcript abundance was decreased (Figure 2B; *p* = 0.014 and *p* = 0.015, respectively) in comparison to the control group, whereas the *CD36* transcript level was unaltered (Figure 2A). The SO group showed an increase in *SCD5* mRNA transcript abundance compared to the control group (Figure 2C; *p* = 0.038).

## 4. Discussion

This study demonstrates, for the first time, that low-level dietary supplementation with RO or SO influences follicle development, hormonal parameters, and the FA profile in juvenile rabbit ovaries. Both oils affected the initial recruitment of ovarian follicles, while only RO influenced the cyclic recruitment of antral follicles. Notably, SO supplementation modified the ovarian FA profile, specifically by increasing the content of MUFAs and decreasing the amount of PUFAs, with the exception of an increased linoleic acid content, which might modulate the local ovarian microenvironment.

In the current study, we observed significant changes in the FA profile of rabbit ovarian tissues following SO supplementation. Among SFAs, SO increased the lauric and myristic acid levels while decreasing the stearic and arachidic acid contents. Evidence from studies on mice, cows, and pigs indicate that SFAs, such as stearic and palmitic acids, exert detrimental effects on ovarian granulosa cells via apoptosis induction [23,32,33]. Herein, we observed a tendency toward a greater number of atretic follicles following SO supplementation; however, these results were not statistically significant. In goats, lauric acid increases plasma E2 concentration during estrus [34]; this is in contrast to our findings. Although we observed a higher lauric acid content in the SO group, the E2 level remained unchanged. We also noted an increase in two MUFAs, namely myristoleic and palmitoleic acids. To explain this, we analyzed the expression of *SCD5* mRNA, which is responsible for the conversion of SFAs into MUFAs [9], and found it to be up-regulated. Similarly, increased mRNA *SCD5* abundance has been reported in the oocytes of dairy cows fed a diet supplemented with palmitoleic acid [35].

Furthermore, in the SO group, we observed decreased levels of several PUFAs: dihomo-γ-linoleic acid/DGLA, arachidonic acid, eicosapentaenoic acid, adrenic acid, and docosapentaenoic acid. The linoleic acid content in this group was greater compared to the control group, reflecting the FA composition of SO, which is particularly rich in this FA [17]. Despite similar chemical compositions, RO did not induce significant changes in the ovarian FA profile. This may be due to the substantially higher FA content in SO compared to RO (e.g., linoleic acid content: 581.45 g/kg in SO vs. 60.15 g/kg in RO; oleic acid content: 207.62 g/kg in SO vs. 24.88 g/kg in RO) [16,17], which might ultimately influence cellular metabolism and FA accumulation. In summary, our findings suggest that the ovarian tissue FA profile established following the SO supplementation might be important in determining ovarian function in juvenile rabbits.

The limiting step in cellular FA utilization is the hydrolysis of triglycerides from circulating lipoproteins by LPL [10]. In vivo studies demonstrated the effect of PUFAs on *LPL* gene expression, which varies depending on tissue type and metabolic demand [36]. Both RO and SO decreased *LPL* mRNA transcript abundance in the rabbit ovary, similar to findings in rats fed high-fat diets containing docosahexaenoic acid or a mix of eicosapentaenoic/docosahexaenoic acids, which showed reduced *Lpl* transcript abundance in retroperitoneal white adipose tissue [34]. The FAs released during triglyceride hydrolysis are subsequently transported into cells via various FA transporters, including CD36 [10]. In general, *CD36* gene expression has been reported to be up-regulated by PUFAs; however, a stronger effect has been attributed to omega-3 than to omega-6 PUFAs [37]. In the current study, neither RO nor SO influenced *CD36* mRNA transcript abundance in the rabbit ovary. These oils are predominantly rich in omega-6 PUFAs, which might not be able to exert a significant effect at the transcriptional level. Collectively, the down-regulation of *LPL* and unchanged *CD36* transcript abundance indicate a lack of RO- and SO-induced effects on the plasma-derived FA uptake by ovarian cells in the rabbit.

Although we did not observe enhanced lipid transporter *CD36* gene expression in the rabbit ovary, both RO and SO supplementation markedly increased plasma P4 concentrations without affecting T levels, suggesting the activation of early steroidogenesis. This is consistent with previous reports indicating a positive effect of UFAs on P4 production. Linoleic acid induces P4 synthesis by increasing the phosphorylation of mitogen-activated protein kinases (ERK1/2) in caprine granulosa cells [21]. Likewise, arachidonic acid has been shown to induce ERK and Akt phosphorylation, leading to elevated P4 production in bovine granulosa cells [38]. Thus, dietary supplementation with RO or SO appears to be sufficient to modulate the initial steps of steroidogenesis despite not increasing lipid delivery to the rabbit ovary.

FAs have been reported to influence ovarian function by affecting steroidogenesis, as well as granulosa cell proliferation and apoptosis, all of which are necessary for proper follicular development [6]. The early stages of ovarian folliculogenesis involve the initial recruitment of primordial follicles from the resting pool and inducing them to grow [39]. In the present study, both RO and SO supplementation reduced the number of primary follicles. This is in accordance with our previous research showing delayed primordial follicle transition into the primary stage following dietary algae (which is rich in omega-3 FAs) and sunflower oil (which is rich in omega-6 FAs) supplementation [27]. In the current study, this shift in follicular dynamics was accompanied by increased plasma AMH concentrations following the administration of both oils. AMH is known to inhibit initial recruitment of primordial follicles in humans [4]; however, its role in rabbit folliculogenesis remains poorly understood. Previous research examining the relationship between AMH concentrations, spay status, pseudopregnancy, and ovarian follicle numbers in female rabbits demonstrated a correlation between serum AMH levels and the number of secondary follicles, which are the primary source of AMH [40]. Our results indicate that RO and SO may affect early follicle growth in juvenile rabbits, potentially through the modulation of AMH concentrations.

Antral follicle development is FSH-dependent. Only follicles responsive to FSH stimulation can enter the final stage of maturation and eventually lead to ovulation. Furthermore, FSH enhances aromatase activity and subsequent E2 biosynthesis within the follicle [41]. In the current study, we found a greater number of antral follicles after RO supplementation, suggesting accelerated folliculogenesis at advanced stages. Additionally, we observed elevated plasma FSH and E2 concentrations, suggesting that RO might stimulate FSH-dependent follicular growth in the juvenile rabbit ovary. Similar results have been reported in studies showing that dietary supplementation with PUFAs enhances follicular maturation and steroidogenesis [11]. Unexpectedly, the addition of SO did not increase the number of antral follicles and E2 production despite the elevated FSH concentrations, indicating a different responsiveness of antral follicles to FSH. Alternatively, differences in the duration and magnitude of FSH stimulation following RO and SO administration may determine the number of follicles undergoing cyclic recruitment [41]. It is also possible that antral follicles are more sensitive to the altered ovarian FA profile caused by SO supplementation, which may modulate the ovarian microenvironment. It should be highlighted that we herein observed non-significant differences regarding the number of primordial and secondary follicles, despite large differences between means. This might be a result of an insufficient sample size to detect relevant differences; thus, conclusions about these variables cannot be clearly stated.

In this study, RO and SO were added to the food pellets, not extracted compounds from the oils. Therefore, we cannot definitely determine which oil’s component (phenolic compounds, tocols, tocopherols, tocotrienols or phytosterols [42]) was responsible for the observed effects or if they were partly due to synergistic actions. Furthermore, the limitation of the study might be the lack of chemical characterization of RO and SO besides FA profile. Although there are many active compounds in RO and SO, based on the literature, we mainly attributed the observed effects to FAs.

## 5. Conclusions

In summary, our study demonstrates, for the first time, the effects of low-level dietary supplementation with RO or SO on folliculogenesis, hormonal parameters, and the FA profile of the juvenile rabbit ovary. Both oils increased AMH concentration, which may contribute to the maintenance of the ovarian reserve by delaying the transition of primordial follicles to the primary stage. In addition, RO increased FSH levels, which might enhance the number of antral follicles undergoing cyclic recruitment and, consequently, E2 concentrations. Interestingly, we observed SO-induced changes in the ovarian FA profile, which may affect the ovarian microenvironment and, subsequently, key ovarian processes such as folliculogenesis and steroidogenesis. However, further mechanistic studies integrating transcriptomic, proteomic, and lipidomic analyses are needed to clarify the molecular pathways linking dietary RO or SO supplementation with ovarian physiology.

## Figures and Tables

**Table 1:** Ingredients and chemical composition of rabbit diets (%).

	Treatment		
	Control	RO	SO
**Ingredient**			
Wheat	29.58	28.58	28.58
Maize	24.50	24.50	24.50
Bran	15.00	15.00	15.00
Sunflower meal	11.00	11.00	11.00
Lucerne meal	10.00	10.00	10.00
Soybean meal	7.00	7.00	7.00
Mineral and vitamin premix	1.50	1.50	1.50
Calcium carbonate	0.80	0.80	0.80
Dicalcium phosphate	0.62	0.62	0.62
Raspberry seed oil	-	1.00	-
Strawberry seed oil	-	-	1.00
**Chemical component**			
Dry matter	89.07	89.12	89.03
Crude ash	7.40	7.27	7.39
Total nitrogen	2.57	2.54	2.50
Total protein	16.42	15.96	16.00
Crude fat	3.90	4.02	4.11
Crude fiber	14.62	14.39	14.96
Estimated digestible energy [MJ/kg]	9.2	9.4	9.5

RO, raspberry seed oil-treated group; SO, strawberry seed oil-treated group. Each treatment (diet) contains (per kilogram): 10.000 IU of vitamin A; 1.500 IU of vitamin D_3_; 30 mg of vitamin E; 7.5 mg of Cu; 50 mg of Fe; 75 mg of Mn; 50 mg of Zn; 1.0 mg of I; 0.2 mg of Se. For estimation of digestible energy (DE), the following equation was used: DE = 12.912 − 0.0236CF + 0.010CP + 0.020EE, where CF is crude fiber, CP is crude protein, and EE is ether extract/crude fat (all in g/kg DM) [26].

**Table 2:** Effects of dietary supplementation with raspberry (RO) or strawberry (SO) seed oil on the number of follicles (mean ± SEM) in the rabbit ovary (*n* = 6/group).

Treatment	Follicle Number (Mean ± SEM)				
	Primordial	Primary	Secondary	Antral	Atretic
**Control**	151.2 ± 57.8	8.5 ± 1.5 ^a^	6.5 ± 2.8	3.66 ± 1.75 ^a^	1.2 ± 0.6
**RO**	117.5 ± 48.0	3.8 ± 2.6 ^b^	13.5 ± 6.5	6.66 ± 3.14 ^b^	1.5 ± 1.2
**SO**	112.2 ± 20.6	4.0 ± 2.0 ^b^	9.8 ± 5.8	5.16 ± 1.16 ^a^	2.5 ± 1.7

Different superscript letters denote statistically significant differences among groups (*p* < 0.05; Dunn’s multiple comparison test).

**Table 3:** The plasma concentrations (mean ± SEM) of progesterone (P4), testosterone (T), estradiol-17β (E2), follicle-stimulating hormone (FSH) and anti-Müllerian hormone (AMH) in rabbits fed a control diet or a diet supplemented with raspberry (RO) or strawberry (SO) seed oil (*n* = 6/group).

	P4 (ng/mL)	T (ng/mL)	E2 (pg/mL)	FSH (mIU/mL)	AMH (pg/mL)
**Control**	0.55 ± 0.24 ^a^	0.52 ± 0.09	112.5 ± 18.2 ^a^	44.85 ± 10.09 ^a^	3484.25 ± 475 ^a^
**RO**	1.32 ± 0.16 ^b^	0.46 ± 0.12	229.65 ± 11.64 ^b^	56.05 ± 3.8 ^b^	4232 ± 711 ^b^
**SO**	1.58 ± 0.2 ^b^	0.45 ± 0.14	89.76 ± 11.9 ^ab^	65.19 ± 10.04 ^b^	4141.5 ± 186 ^b^

Different superscript letters denote statistically significant differences among groups (*p* < 0.05; Dunn’s multiple comparison test).

**Table 4:** Effects of dietary supplementation with raspberry (RO) or strawberry (SO) seed oil on the fatty acid profile (mean ± SEM) in the rabbit ovary (*n* = 4/group).

	Fatty Acids (%)	Treatment		
		Control	RO	SO
**Saturated fatty acids (SFAs)**	10:0 (decanoic acid)	0.12 ± 0.012	0.14 ± 0.007	0.15 ± 0.048
	12:0 (lauric acid)	0.27 ± 0.38 ^a^	0.36 ± 0.076 ^ab^	0.96 ± 0.016 ^b^
	14:0 (myristic acid)	1.34 ± 0.131 ^a^	1.77 ± 0.07 ^ab^	3.05 ^b^ ± 0.025
	15:0 (pentadecanoic acid)	0.47 ^a^ ± 0.023	0.45 ^a^ ± 0.009	0.54 ± 0.008 ^a^
	16:0 (palmitic acid)	22.08 ± 0.055	22.69 ± 0.673	22.74 ± 1.624
	17:0 (heptadecanoic acid)	0.73 ± 0.037	0.63 ± 0.008	0.60 ± 0.016
	18:0 (stearic acid)	14.29 ± 0.609 ^a^	11.98 ± 0.22 ^ab^	7.74 ± 0.37 ^b^
	20:0 (arachidic acid)	0.22 ± 0.011 ^a^	0.17 ± 0.007 ^ab^	0.13 ± 0.012 ^b^
**Monounsaturated fatty acids (MUFAs**)	14:1 (myristoleic acid)	0.05 ± 0.002 ^a^	0.11 ± 0.006 ^ab^	0.25 ± 0.025 ^b^
	16:1 *n-9* (palmitoleic acid)	0.59 ± 0.046	0.60 ± 0.036	0.44 ± 0.11
	16:1 *n-7* (palmitoleic acid)	0.88 ± 0.029 ^a^	1.87 ± 0.145 ^ab^	2.43 ± 0.13 ^b^
	17:1 (heptadecenoic acid)	0.23 ± 0.008	0.25 ± 0.027	0.26 ± 0.037
	18:1 *n-9* (oleic acid)	22.57 ± 1.18	25.04 ± 0.662	26.44 ± 0.803
	18:1 *n-7* (cis-vaccenic acid)	1.37 ± 0.064 ^ab^	1.40 ± 0.022 ^a^	1.05 ± 0.046 ^b^
	20:1 (eicosenoic acid)	0.42 ± 0.019	0.44 ± 0.036	0.32 ± 0.015
**Polyunsaturated fatty acids (PUFAs)**	18:2 *n-6* (linoleic acid)	13.94 ± 0.884 ^a^	16.36 ± 1.141 ^ab^	25.96 ± 2.128 ^b^
	18:3 *n-6* (γ-linolenic acid/GLA)	0.16 ± 0.002	0.15 ± 0.011	0.08 ± 0.001
	18:3 *n-3* (α-linolenic acid/ALA)	0.72 ± 0.13	0.88 ± 0.023	1.66 ± 0.15
	CLA (rumenic acid)	0.03 ± 0.009	0.03 ± 0.007	0.03 ± 0.06
	20:2 (eicosadienoic acid)	0.71 ± 0.095	0.66 ± 0.016	0.36 ± 0.06
	20:3 *n-6* (dihomo-γ-linoleic acid/DGLA)	1.46 ± 0.165 ^a^	1.15 ± 0.068 ^ab^	0.40 ± 0.046 ^b^
	20:4 *n-6* (arachidonic acid)	10.70 ± 0.865 ^a^	7.88 ± 0.518 ^ab^	2.73 ± 0.169 ^b^
	20:4 *n-3* (eicosatetraenoic acid)	0.04 ± 0.002	0.05 ± 0.002	0.03 ± 0.003
	20:5 *n-3* (eicosapentaenoic acid/EPA)	0.26 ± 0.042 ^a^	0.12 ± 0.008 ^ab^	0.01 ± 0.001 ^b^
	22:4 *n-6* (adrenic acid)	3.78 ± 0.404 ^a^	2.74 ± 0.182 ^ab^	1.01 ± 0.102 ^b^
	22:5 *n-6* (osbond acid)	1.07 ± 0.015	0.80 ± 0.078	0.27 ± 0.014
	22:5 *n-3* (docosapentaenoic acid)	1.09 ± 0.085 ^a^	0.85 ± 0.057 ^ab^	0.26 ± 0.007 ^b^
	22:6 *n-3* (docosahexaenoic acid/DHA)	0.35 ± 0.035	0.38 ± 0.089	0.06 ^a^± 0.006
	Total	99.95 ± 0.007	99.95 ± 0.005	99.98 ± 0.002

Different superscript letters denote statistically significant differences among groups (*p* < 0.05; Dunn’s multiple comparison test).

## Data Availability

The data that support the findings of this study are available from the corresponding author upon request.
